# In situ facile green synthesis of Ag–ZnO nanocomposites using *Tetradenia riperia* leaf extract and its antimicrobial efficacy on water disinfection

**DOI:** 10.1038/s41598-022-19403-1

**Published:** 2022-09-13

**Authors:** Stanslaus G. Mtavangu, Revocatus L. Machunda, Bart van der Bruggen, Karoli N. Njau

**Affiliations:** 1 Department of Chemical Engineering, Faculty of Engineering Sciences, KU Leuven, Celestijnenlaan 200F, 3001 Leuven, Belgium; 2 School of Materials Energy Water and Environmental Sciences, Nelson Mandela African Institution of Science and Technology, PO Box 447, Arusha, Tanzania; 3 Department of Chemistry, Dar es Salaam University College of Education, P.O. Box 2329, Dar es Salaam, Tanzania; 4 Faculty of Engineering and the Built Environment, Tshwane University of Technology, Private Bag X680, Pretoria, 0001 South Africa

**Keywords:** Materials science, Nanoscience and technology

## Abstract

In this work, Ag–ZnO nanocomposites were prepared by a green synthesis route using aqueous leaf extract of *Tetradenia riperia* and investigated for antibacterial activity against *Escherichia coli* and *Staphylococcus aureus.* To optimize the synthesis of the Ag–ZnO, the effects of precursor concentrations, pH, and temperatures were studied. The Ag–ZnO nanocomposites were characterized by XRD, ATR-FTIR, FESEM, and TEM. Results show that the concentration of 8% Ag, the temperature of 80 °C, and a pH of 7–8 were optimal for the synthesis of Ag–ZnO nanocomposites. The XRD analysis showed the decrease in particle size of Ag–ZnO from 23.6 to 14.8 nm with an increase in Ag concentrations, which was further supported by FESEM analysis. TEM image of 8% Ag provides more information on the coexistence of Ag on ZnO where an average particle size of 14.8 nm was determined. The ATR-FTIR analysis confirmed the presence of phenolic compounds, which work as reducing and stabilizing agents. The antimicrobial activity results show that Ag–ZnO nanocomposite demonstrated a higher antimicrobial potency on *E. coli* than on *S. aureus.* Therefore, *Tetradenia riperia* leaf extract is a viable route for the synthesis of Ag–ZnO nanocomposites to be used for various applications, including water disinfection.

## Introduction

The sustainable development goals (SDGs 2030), enshrine through goal six a declaration that clean water and sanitation are pivotal to human development^1^. This is because waterborne infections caused by microorganisms are a leading cause of death worldwide^2,3^. Therefore, the need to find affordable, efficient, versatile, and sustainable technologies to control and eliminate microbes from drinking water is inevitable^3^. The implementation of water disinfection technologies to eliminate pathogens in centralized and some decentralized water treatment systems is achieved through conventional methods that include chlorination, ozonation, and ultraviolet treatment^3,4^. However, chlorination is limited by the formation of toxic byproducts, while ozonation and ultraviolet offer no protection against recontamination in the distribution systems^4,5^. Therefore, this necessitates the deployment of alternative treatment technologies.

In recent years, nanotechnology through nanomaterials has emerged as an effective and versatile tool for water disinfection through its ability to cope with resistant pathogens. Normally, microbes adapt to drug resistance by protecting themselves against all odds and mutating to enable them to survive and reproduce even in harsh environments^6^. Nanomaterials have been studied as a potential solution to water disinfection challenges^4,7^ because pathogens find it hard to acquire resistance to nanoparticles that target multiple bacterial components, compared to the use of bulk materials during conventional treatment techniques. The application of silver metal as an antimicrobial agent has been documented since ancient times^8^. Since the nineteenth century, silver ions have been associated with bactericidal effects^9^. Recently, the prevalence of antimicrobial drug resistance to antibiotics has been increasing therefore, the use of silver as a disinfectant is inevitable. Silver is now used in consumer products such as textiles, cosmetics, and medical instruments^10–12^ in the form of nanoparticles, which are prepared by the chemical reduction of silver salts^13^. Furthermore, studies have documented its potential in water disinfection^14–17^. Therefore, silver is a fascinating and promising candidate to be explored due to its inhibitory and antibacterial capabilities among various metal nanoparticles^6,18^. However, silver nanoparticles can aggregate when their size is much reduced, which limits their chemical and antimicrobial properties. Therefore, to address this challenge, silver can either be capped with polymers to make polymeric nanocomposites^6,18^ or with a layer of metal oxide, like magnesium oxide, calcium oxide, and zinc oxide, to form a core–shell shape that provides a high surface area^6^. Moreover, when nanomaterials are synergized, hybrid nanocomposites that are more powerful than the individual nanoparticles can be produced; these are expected to combine the properties of the constituent elements^19^.

Various studies have indicated the antimicrobial efficacy of ZnO nanoparticles against both gram-positive and gram-negative bacteria^20–22^. The antibacterial properties of ZnO nanoparticles have been geared toward various applications that include controlling foodborne pathogens^23^, and waterborne pathogens from drinking water^4^, due to their high biocompatibility and bactericidal effects^19^. ZnO has lately been acknowledged by the United States Food and Drug Administration (21CFR182.8991) (FDA, 2011) as a safe material^6,24^ with a short lifespan in the body that lasts for only some hours^25^. ZnO nanoparticles suppress bacterial growth by a variety of processes, including cell penetration, electrostatic adhesion to the bacterial surface, and the formation of reactive oxygen species^26^. In this regard, ZnO nanoparticles can be considered viable and effective nanomaterials to be used for protection against antibacterial infections^27^. Therefore, synergizing Ag nanoparticles with ZnO nanoparticles will yield a nanocomposite material with stronger antibacterial properties for both gram-positive and gram-negative bacteria^18^.

Metal and metal oxide nanoparticles have gained much attention as antibacterial materials because of their high reactivity, which is related to their very high surface area to volume ratio^28^. As a consequence, the potential of Ag–ZnO nanocomposites can be harnessed and applied in the field of environmental health for the development of antimicrobial compounds in view of water disinfection. In order to exploit synergies, different alloys have been synthesized and shown a superior antimicrobial effect during water disinfection against bacterial drug resistance^29,30^. The Ag–ZnO nanocomposites display a fascinating efficiency ascribed to their plasmonic properties and interfacial electron exchange process. Furthermore, when compared to pure ZnO nanoparticles, the efficacy of Ag–ZnO nanocomposites has been assessed based on their silver and zinc ion release in aqueous solution, stability, reusability, and lasting bactericidal effect, which are all significantly higher^19^.

Several methods have been employed for the synthesis of Ag–ZnO nanocomposites, which include co-precipitation^31^, sol–gel^32^, and hydrothermal synthesis^33^, to mention just a few. However, the application of high temperatures and pressures, longer reaction times, and the generation of chemical waste^34–37^ hamper their application in large-scale production. Therefore, the development of an environmentally benign technology in material synthesis is inevitable. The green synthesis of nanomaterials by using natural biogenic materials such as fungi, bacteria and plant parts is growing^19,27,34,38–40^. The literature survey reports the green synthesis of Ag–ZnO nanocomposites from different medicinal plant parts such as *moringa oleifera* seeds^41^, *Azadirachta indica* leaf extract^42^, *Zingiber zerumbet* rhizome^43^, potato peeland^44^, *Murraya koenigii* and *Zingiber officinale* extracts^19^ and guajava leaves^45^. In green synthesis, the properties of nanomaterials are influenced by the plant extract because each plant extract contains a specific concentration and combination of biomolecules^46^. Therefore, nanomaterials synthesized from different plant exhibit different antimicrobial efficacy. This motivates more exploration of plant species for the synthesis of nanomaterials^18,42,47^.

In this regard, the use of plant extracts via biosynthetic pathways is regarded as the most viable approach for the synthesis of nanomaterials. The plant extracts contain active compounds such as alkaloids, flavonoids, proteins, tannins, terpenoids, saponin, and polyphenols^34,42,43,48,49^. These phytocompounds act as reducing agents for metal ions (natural weak base) and capping agents for the nuclei to prevent the agglomeration of nanoparticles^34,35^, thus improving their reactivity. To harness the potential of the aforementioned phytocompounds, this study reports a green route for the synthesis of Ag–ZnO from silver and zinc salts using leaf extract from the medicinal plant *Tetradenia riperia* (TR). *Tetradenia riperia* (TR) belongs to the family *Lamiaceae* and is found abundantly in the northern regions of Tanzania. The plant has been used for medicinal purposes to treat diarrhea, indigestion, constipation, malaria, coughs, and sore throats by ethnic groups that include Meru, Maasai, Pare, and Chaga from the northern regions of Tanzania^50^. TR leaves are also being reported to show antibacterial, anti-inflammatory, and anticancer properties^51,52^. Phytochemical analysis revealed the presence of active compounds such as alkaloids, flavonoids, phenols, saponins, tannins, steroids, and reducing sugar^34,50,52^, which can be used as reducing and stabilizing agents in the synthesis of nanoparticles^35^. The spatial distribution of *Tetradenia riperia* has been documented in various areas of African countries such as Rwanda^53^, Madagascar^54^, South Africa^55^, Uganda^56^ and Tanzania^57^ in which its medicinal potential has been attributed to abundant phytocompounds with antimicrobial effects^51^. Literature has reported the use of *Tetradenia riperia* leaves in the synthesis of various nanoparticles such as silver^58,59^. To the best of our knowledge, no study has reported on the synthesis of Ag–ZnO nanocomposites by using the aqueous leaves extract of *Tetradenia riperia*. Therefore, this work presents a novel *Tetradenia riperia* leaves extracts-based Ag–ZnO nanocomposites and evaluates its antibacterial activity against antibiotic-resistant bacterial strains. The Ag–ZnO nanocomposite will later be used for the development of water filters for the disinfection of water at the point of use.

## Materials and methods

### Plant materials

*Tetradenia riperia* leaves were collected from Musini village in Uru, North Moshi-Kilimanjaro region, and Kilala village, along the slopes of Mount Meru in the Arusha region, Tanzania. The identification of *Tetradenia riperia* was done by Njau et al.^60^. A voucher specimen (reference no. EN 2980/2013) has been deposited at National Herbarium of Tanzania (NHT) at Tropical Pesticides Research Institute (TPRI)-Arusha. The collection of the plant materials complied with relevant institutional, national, and international guidelines and legislation.

### Chemicals

Analytical grade silver nitrate (AgNO_3_) (99%), zinc nitrate hexahydrate (Zn(NO_3_)_2_.6H_2_O) (95%) and sodium hydroxide NaOH (98%) were purchased from Sigma-Adrich Chemicals (Germany).

### Preparation of *Tetradenia riperia* (TR) leaves extract

**Figure 1:**
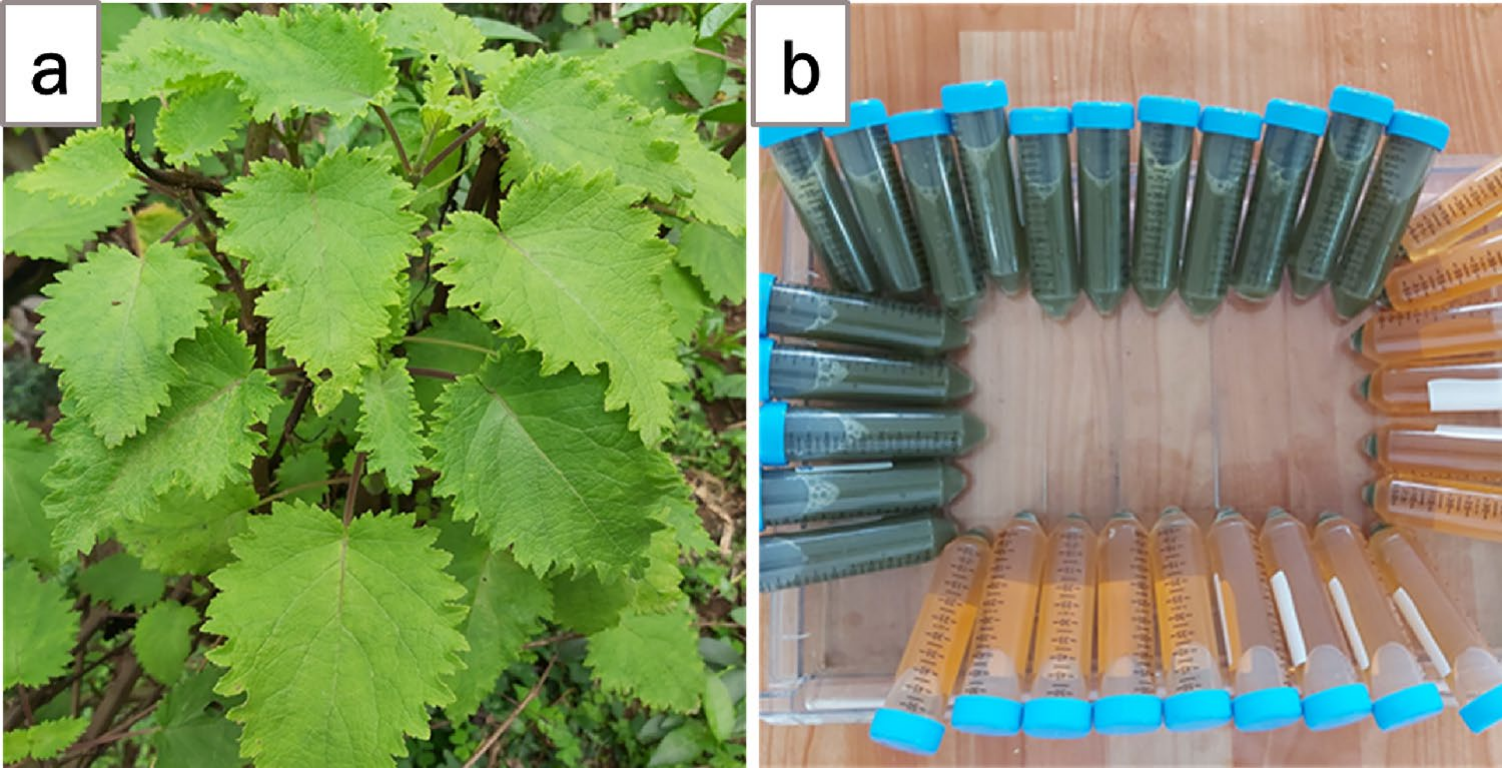
Image of (**a**) *Tetradenia riperia* plant leaves (**b**) uncentrifuged and centrifuged aqueous leaves extract.

The collected leaves were washed several times with tap water followed by double distilled water (DD) to remove any dust particles from the surface. Then fresh leaves were chopped into small pieces and ground by using an electric mortar and blender into fine particles. About 20 g of fresh, ground *Tetradenia riperia* leaves were mixed in 100 ml of double-distilled water followed by shaking on a mechanical shaker for 24 h to allow effective extraction of phytocompounds from TR leaves. The obtained aqueous extract was centrifuged at 4000 rpm for 15 min, then the supernatant (brown color) was filtered using Whatman No.1 filter paper and stored at 4 °C ready for further use as a reducing and stabilizing agent during the green synthesis of Ag–ZnO nanocomposites (Fig. 1). The pH of *Tetradenia riperia* aqueous leaves extract was 5.10.

### Synthesis of Ag–ZnO nanocomposites and ZnO nanoparticles

#### Biosynthesis of ZnO nanoparticles

The synthesis of ZnO nanoparticles was carried out based on the literature^27^ with modification. For the synthesis of ZnO NPs, 2.9748 g of ZnO (NO_3_)_2_·6H_2_O was dissolved in 100 ml of *Tetradenia riperia* leaf extract in a 250 ml Erlenmeyer flask, then stirred for 30 min. This lowered the pH of the media to 4.9. Then 0.1 M NaOH was added dropwise while stirring to adjust the pH of the media to 7–8. The batch was set under continuous stirring on magnetic stirring plate at room temperature (30 °C) for 3 h. The resultant mixture was incubated to age for 24 h, followed by centrifugation at 4000 rpm for 15 min. The supernatant was discarded, and the precipitate was redispersed in an ethanol–water mixture at a ratio of 1:1 (v/v), and then recentrifuged. The centrifugation and redispersion processes were repeated three times. Similar procedures were adopted for the batches prepared at 60 and 80 °C. The purified precipitates were dried at 80 °C in a hot air oven for 4 h, then calcined at 450 °C for 2 h at a heating rate of 5 °C/min. The white-coloured residues formed were ground into powder by using a Hargett mortar and pestle, then stored in an airtight container ready for further analysis and use.

#### Biosynthesis of Ag–ZnO nanocomposite

The Ag–ZnO nanocomposites were synthesized using a green synthesis method, adopted from the literature^41^ with slight modification for what concerns the synthesis temperature, reaction time, and the ageing time. Herein, about 2.9748 g of Zn (NO_3_)_2_·6H_2_O was slowly dispersed in 90 ml of *Tetradenia riperia* leaf extract for 30 min while magnetic stirring, then 10 ml of 0.01 M AgNO_3_ solution was added while stirring for another 30 min at room temperature (30 °C) to achieve a final concentration of 0.1 M Zn (NO_3_)_2_·6H_2_O and 1 mM AgNO_3_. This lowered the pH of the media from 5.10 to 4.46. The pH was then adjusted to 7–8 by adding 0.1 M NaOH solution dropwise while continuously stirring. During the reaction, a color change was observed, where the brown color of the *Tetradenia riperia* extract changed to pale yellow after the addition of zinc nitrate salt, then to dark yellow after the addition of AgNO_3_ solution, signifying the reduction of Zn^2+^ and Ag^+^ ions, and later to dark greenish yellow after the addition of NaOH solution. Three batches were prepared in this way at three different synthesis temperatures; room temperature (30 °C), 60 and 80 °C in an oil bath fitted out with magnetic stirring hot plates at 400 rpm for 3 h. The batches were allowed to cool and age for 24 h at room temperature; thereafter, they were centrifuged at 4000 rpm for 15 min; the precipitates were washed again three times with a mixture of ethanol–water prepared at a 1:1 ratio (v/v) and then re-centrifuged at 4000 rpm for 15 min. The obtained precipitates were oven dried at 80 °C for 4 h, followed by calcination at 450 °C for 2 h at a heating rate of 5 °C/min. The obtained nanohybrid crystals were ground into powder by using a Hargett mortar and pestle, then stored in an airtight container ready for characterization and use. Similar procedures were adopted in synthesizing Ag–ZnO nanocomposites with different dopant concentrations of silver nitrate calculated according to the molar ratio of [Ag^+^]/[Zn^2+^] = 1%, 3%, 5%, and 8%, and their corresponding nanocomposites (NC) were marked as NC1, NC3, NC5, and NC8, respectively.

### Material characterization

The crystalline phase and purity of the synthesized Ag–ZnO nanocomposites and ZnO nanoparticles were examined by a Bruker BV 2D PHASER Best Benchtop (PANalytical BV, Amsterdam, the Netherlands) X-ray diffraction (XRD) analyzer with reflection geometry at 2θ values (10°–90°) with a step size of 0.005°, working with a Cu Kα radiation source (λ = 0.15406 nm) at 50 kV and 30 mA. The functional groups were ascertained by attenuated total reflection-Fourier transform infrared (ATR-FTIR) spectroscopy (Bruker Optic GmbH 2011 (alpha model, Laser class 1) in transmittance mode and a spectral range of 4000–400 cm^−1^ with a spectral resolution of 2 cm^−1^. The morphology and elemental composition of nanomaterials were examined by Zeiss Ultra Plus 55 field emission scanning electron microscopes (FE-SEM) equipped with an energy dispersive X-ray (EDX) and a high-resolution transmission electron microscope (HRTEM FEI Tecnai-F30; Akishima-shi, Japan) operated at 1.0 kV. Ultraviolet–visible (UV–Vis) absorption spectra were recorded on a spectrophotometer (UVmini-1240 Shimadzu, Japan), in the wavelength range of 300–800 nm.

### Antibacterial assay

The antibacterial activity of the synthesized ZnO nanoparticles and Ag–ZnO nanocomposites was assessed against gram-positive (*Staphylococcus aureus*- ATCC 6538P) and gram-negative (*Escherichia coli*-ATCC 9677) strains using the disc diffusion method^47^. The prepared and sterilized nutrient agar media (15–20 ml) was poured into the sterilized petri dishes and allowed to solidify. After solidification of the nutrient agar medium, each bacterial strain was inoculated onto individual agar dishes and spread uniformly by using a sterilized swab. The sterilized absorbent discs of about 6 mm were soaked in a colloidal solution of ZnO and Ag–ZnO (1, 3, 5, and 8% Ag) with different concentrations (minimum inhibitory concentration-MIC) (0.5, 1.0, and 1.5 mg/ml). The soaked discs were placed on the inoculated Petri dishes along with Ciprofloxacin discs as standard and *Tetradenia riperia* extract discs as control. Each plate is comprised of three test discs, one standard disc, and one control disc. Thereafter, all dishes were incubated at 37 °C for 24 h. The antimicrobial activity of ZnO nanoparticles and Ag–ZnO nanocomposites was determined by measuring the zone of inhibition (ZOI), which appeared as clear areas around the discs.

## Results and discussion

### X‑ray diffraction

**Figure 2:**
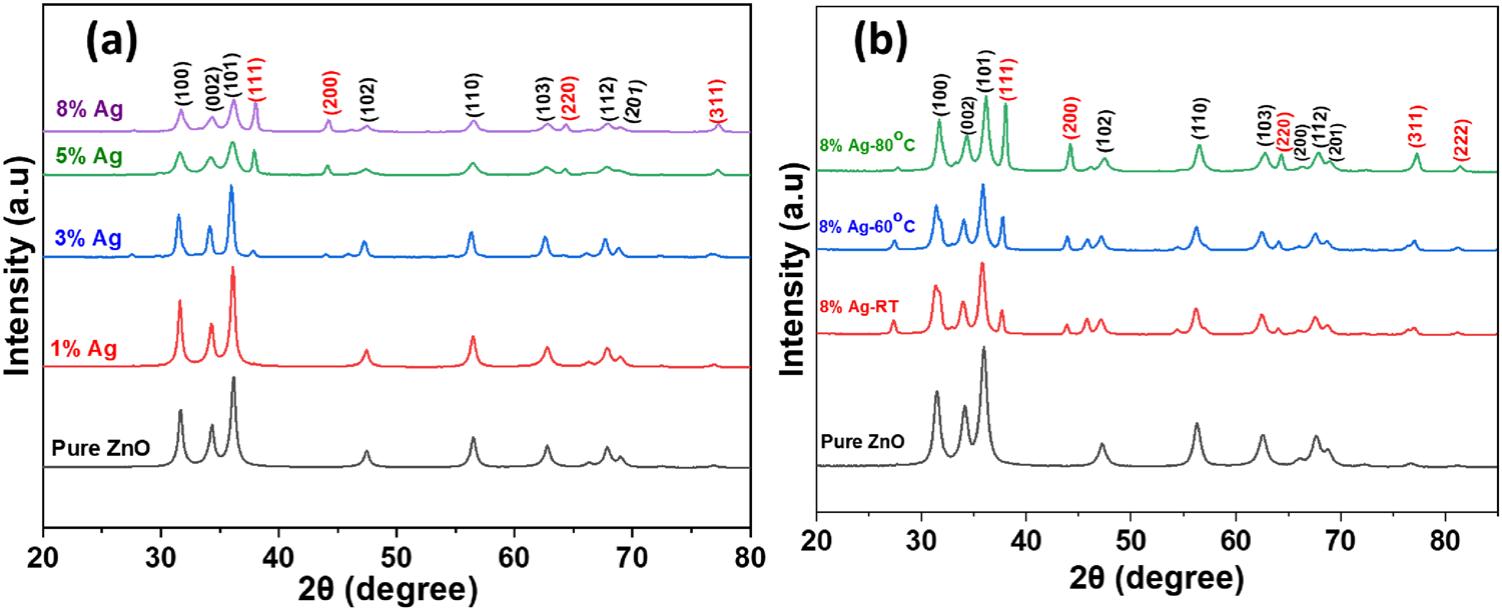
XRD patterns (**a**) pure ZnO and Ag–ZnO nanocomposites at different Ag content (1% Ag, 3% Ag, 5% Ag, 8% Ag) and (**b**) pure ZnO and Ag–ZnO nanocomposites at different synthetic temperatures (RT (30 °C), 60 °C and 80 °C) (The characteristic peaks for Ag are in red hkl).

Figure 2a shows XRD patterns of pure ZnO and Ag–ZnO nanocomposites with various Ag contents (1, 3, 5, and 8%). The pure ZnO patterns display diffraction peaks positions at 2*θ* values of 31.65°, 34.31°, 36.14°, 47.44°, 56.50°, 62.77°, 67.85°, and 69.01° which are indexed to the crystal planes of (100), (002), (101), (102), (110), (103), (112), and (201) of hexagonal wurtzite structures, respectively of ZnO nanoparticles (JCPDS file no. 36–1451)^44,61^. Furthermore, the Ag–ZnO nanocomposite exhibits four additional peaks upon doping with different concentrations of Ag salts, at 38.36°, 44.20°, 64.55°, and 77.74° which match with (111), (200), (220), and (311) planes, respectively^62^. These peaks are indexed to a face center cubic (fcc) structure of Ag nanoparticles according to JCPDS, card No. 04-0783^63,64^, confirming the presence of Ag nanoparticles in the composite^44^. The peaks intensity gradually increases with an increase in the Ag content, which signifies the successful formation of Ag nanoparticles on the ZnO surface. While the Ag peaks intensity increases, as the Ag concentration increases, the peak intensity of ZnO decreases, implying a decrease in the crystallinity and particle size in Ag–ZnO nanocomposites^65^. The average grain sizes (D) calculated from Scherrer's Eq. (1) for pure ZnO, 1%, 3%, 5% and 8% Ag–ZnO nanocomposites were 10.8, 23.6, 20.3, 15.4 and 14.8 nm, respectively. The increase in crystal size from pure ZnO to 1% Ag–ZnO (NC1) might be attributed to the Ag anchoring on the ZnO surface^44^. However, a subsequent decrease in the average crystal size of Ag–ZnO with an increase in Ag content might be ascribed to the dispersion of Ag nanoparticles in or near the boundary of ZnO lattice, which limits the alliance and diffusion of ZnO, thus hinders the growth of the nanocomposite. A similar scenario was reported elsewhere^65,66^. No further peaks were identified, suggesting no linked work function between Ag and ZnO and thus the synthesized Ag–ZnO nanocomposite is pure. This provides insights that doping Ag on the surface of ZnO-NPs was more successful than into the ZnO lattice, attributed to the larger ionic radius for Ag^+^ (1.26 Å) compared to Zn^2+^(0.74 Å)^44^. Furthermore, an increase in the synthetic temperature from 30 to 80 °C exhibited additional peaks in the Ag–ZnO nanocomposite (Fig. 2b). Apart from, hexagonal wurtzite structure peaks of ZnO nanoparticle, new five peaks at 38.36°, 44.20°, 64.55°, 77.74°, and 81.74° indexed to the crystal planes of (111), (200), (220), (311) and (222) respectively were observed. These peaks correspond to the face center cubic (fcc) structure of Ag nanoparticles according to JCPDS, card No. 04-0783^63^, confirming the existence of metallic Ag in Ag–ZnO nanocomposites. The intensity and sharpness of Ag peaks gradually increase with the increase in the synthesis temperature, providing insight that more metallic Ag forms in the matrix at higher temperatures. This might be ascribed to the high nucleation rate leading to the formation of smaller crystalline nuclei^67^) and distinct nanoparticles.

1 $$\mathrm{D}=\frac{\mathrm{K\lambda }}{{\upbeta }_{2\uptheta }\mathrm{Cos\theta }}$$

where D is the average crystallite size in nm, K is the Scherrer constant; equal to 0.9, λ is the specific wavelength of X-ray used (0.154 nm), θ is the diffraction Bragg angle and β_2θ_ is the angular width in radians at an intensity equal to full width and half maximum.

### Optical properties

#### ATR-FTIR study

**Figure 3:**
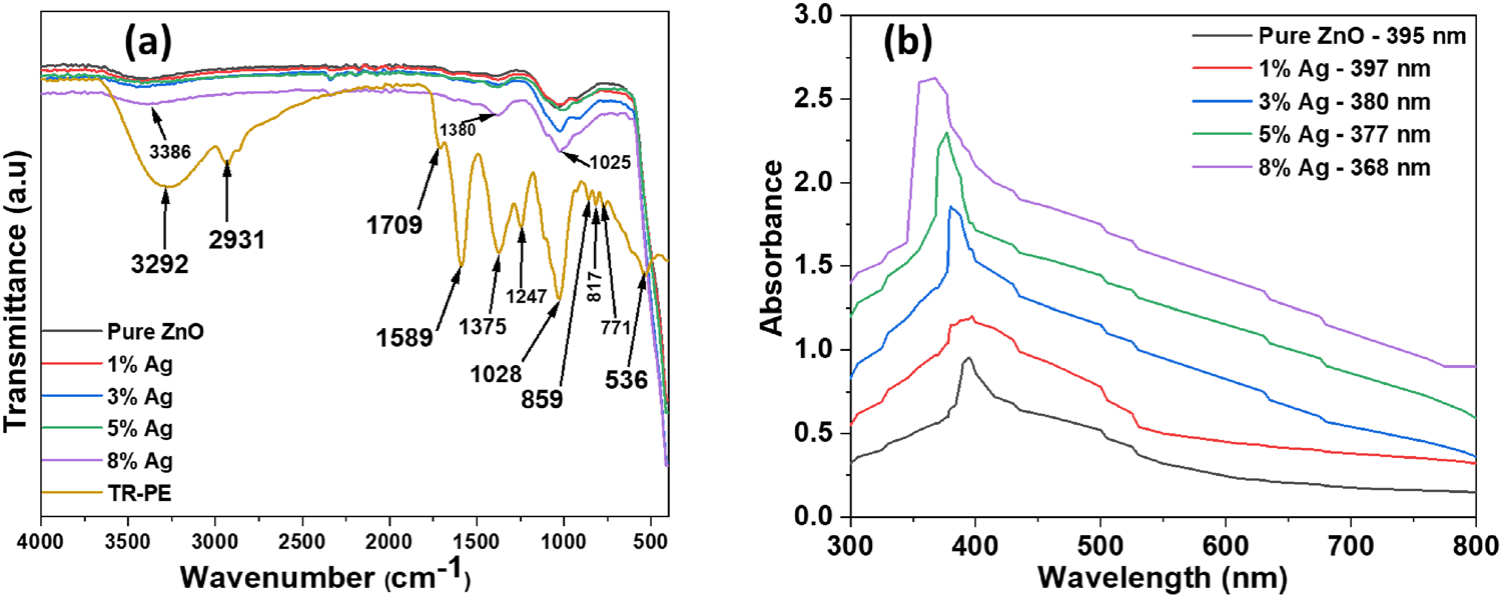
ATR-FTIR spectra of (**a**) Tetradenia riperia plant extract (TR-PE), pure ZnO and Ag–ZnO nanocomposites at different Ag concentrations (**b**) UV–Vis spectra pure ZnO-NPs and Ag–ZnO nanocomposites doped with different concentrations of Ag ions.

Figure 3a shows the FTIR spectrum of the active biomolecules from *Tetradenia riperia* leaves that work as reducing and capping agent during the biosynthesis of nanomaterials. The absorption peaks at 3292, 2931, and 1709 cm^−1^ correspond to the O–H stretching, C–H sp^3^ stretching and C=O stretching mode of the carbonyl group respectively, suggesting the presence of alcohol, polyphenols, amides, esters and acids that signifies the availability flavonoids, saponins, tannins, alkaloids, and reducing sugars^34–36,68^. The adsorption peaks at 1589, and 1375 cm^−1^ are due to the C=C stretching of alkene present in the aromatic ring structure and C–C stretching from the flavonoids respectively^34,69^. The adsorption peaks at around 1247 and 1028 cm^−1^ are assigned to C–N stretching groups of amines^35,49,70^, whereas the 859 cm^−1^ peak is attributed to N–H bending vibration of amine^35^. Moreover, the absorption bands at 817 and 772 cm^−1^ are due to C=C bending for vinylidene and trisubstituted alkene, whereas, band at 536 cm^−1^ represents the aromatic C–H out of plane bending in polyphenols^68^. The amine C–N stretching and N–H bending from the TR extract confirm the presence of alkaloids^36,39^. Alkaloid compounds work as weak base due to presence of nitrogen atoms in cyclic rings which provide electrons pairs to react with water molecules to produce ^−^OH ions which hydrolyses or reduces the metal ions^34,36,38,39^. On the other hand, the ^−^OH bending and ^−^OH stretching depict the presence of flavonoids, tannins, and saponins, in TR extract which act as capping agents to prevent agglomeration and thus control the particle size^34,36,39,71^. During nanoparticles formation the ^−^OH hydrophilic head of phytocompounds interact with metal ions whereas hydrophobic part provides a steric hindrance that prevent the agglomeration of nanoparticles^36,72^. Apart from the OH groups, studies have shown that the C=C and C=O groups from the phytocompounds can work as a capping agents^48^. Furthermore, it has been observed that some TR extract peaks (3292, 2931, 1709, 1589, 1247,859, 817, and 771 cm^−1^) disappeared after the formation of ZnO nanoparticles and Ag–ZnO nanocomposites, which provides an insight of the participation of biomolecules in the reduction and stabilization of the nanomaterials. However, a broad peak at 3386 cm^−1^ was observed, representing a characteristic peak of OH groups of the absorbed water on the surface of nanoparticles. Moreover, broad peaks at 1380 and 1025 cm^−1^ for polyphenols from TR leaf extract remained on the surface of the ZnO and Ag–ZnO nanocomposite, providing further evidence on the involvement of *Tetradenia riperia* phytocompounds in the formation of nanoparticles by this green route. The mechanisms for biosynthesis of ZnO nanoparticles and Ag–ZnO nanocomposites using TR leave extract can be presented by the following reactions.

$$ \begin{aligned} & {\text{R}} -{\text{ NH}} - {\text{CH}}_{{\text{3(aq)}}} {\text{ + H}}_{{2}} {\text{O}}_{{\text{(l)}}} \to {\text{R  }}-^{ + } {\text{NH}}_{{2}} {\text{ - CH}}_{{\text{3(aq)}}} {\text{ + OH}}^{{-}}_{{\text{(aq)}}} \\ & {\text{AgNO}}_{{\text{3(s)}}} {\text{ + H}}_{{2}} {\text{O}}_{{\text{(l)}}} \to {\text{Ag}}^{ + }_{{\text{(aq)}}} {\text{ + NO}}_{{\text{3(aq)}}}^{ - } {\text{ + H}}_{{2}} {\text{O}}_{{\text{(l)}}} \\ & {\text{Zn(NO}}_{{3}} {)}_{{2}} \cdot {\text{6H}}_{{2}} {\text{O}}_{{\text{(s)}}} {\text{ + 2H}}_{{2}} {\text{O}}_{{\text{(l)}}} \to {\text{Zn}}^{{2 + }}_{{\text{(aq)}}} {\text{ + 2NO}}_{{\text{3(aq)}}}^{ - } {\text{ + 8H}}_{{2}} {\text{O}}_{{\text{(l)}}} \\ & {\text{Zn}}^{{2 + }}_{{\text{(aq)}}} {\text{ + 2OH}}^{ - }_{{\text{(aq)}}} \to {\text{Zn(OH)}}_{{\text{2(s)}}} \\ & {\text{Zn(OH)}}_{{\text{2(s)}}} \mathop{\longrightarrow}\limits_{\Delta }^{}{\text{ZnO}}_{{\text{(s)}}} {\text{ + H}}_{{2}} {\text{O}}_{{\text{(g)}}} \\ & {\text{Ag}}^{ + }_{{\text{(aq)}}} {\text{ + OH}}^{ - }_{{\text{(aq)}}} \to {\text{Ag}}_{{2}} {\text{O}}_{{\text{(s)}}} {\text{ + H}}_{{2}} {\text{O}}_{{\text{(l)}}} \\ & {\text{2Ag}}_{{2}} {\text{O}}_{{\text{(s)}}} \mathop{\longrightarrow}\limits_{\Delta }^{}{\text{4Ag}}_{{\text{(s)}}} {\text{ + O}}_{{\text{2(g)}}} \\ & {\text{Ag}}^{ + }_{{\text{(aq)}}} {\text{ + Zn}}^{{2 + }}_{{\text{(aq)}}} {\text{ + 3OH}}^{ - }_{{\text{(aq)}}} \to {\text{AgZn(OH)}}_{{\text{3(s)}}} \\ & {\text{AgZn(OH)}}_{{\text{3(s)}}} \mathop{\longrightarrow}\limits_{\Delta }^{}{\text{AgZnO}}_{{\text{(s)}}} {\text{ + H}}_{{2}} {\text{O}}_{{\text{(g)}}} \\ \end{aligned} $$

#### UV–Vis spectrum

Figure 3b shows the UV–Vis absorption spectra of pure ZnO nanoparticles and 1% Ag, 3% Ag, 5% Ag, and 8% doped ZnO nanocomposites. Results show that pure ZnO nanoparticles showed an absorption peak at 395 nm, due to the excitonic absorption, while the Ag-doped ZnO nanocomposites with 1%, 3%, 5%, and 8% Ag content exhibited absorption peaks attributed to surface plasmon resonance at 397, 380, 377 and 368 nm, respectively. The increase in peak from 395 to 397 nm (redshift) can be ascribed to the increase in particle size attributed to Ag doping on the ZnO matrix^73^. This increases the distance between the valence bands, which lowers the frequency of electromagnetic emission. Furthermore, the intensity of the absorption of doped ZnO decreases from 397 to 368 nm with an increase in Ag content (blue shift). This can be explicated by a reduction in particle size of the Ag–ZnO nanocomposites attributed to anchored Ag nanoparticles and the formation of smaller nuclei on the ZnO surface^43,74^, which obstructs the movement and diffusion of ZnO as evinced by the XRD study. Similar results were also reported elsewhere^43^.

### Morphological study of nanomaterials

**Figure 4:**
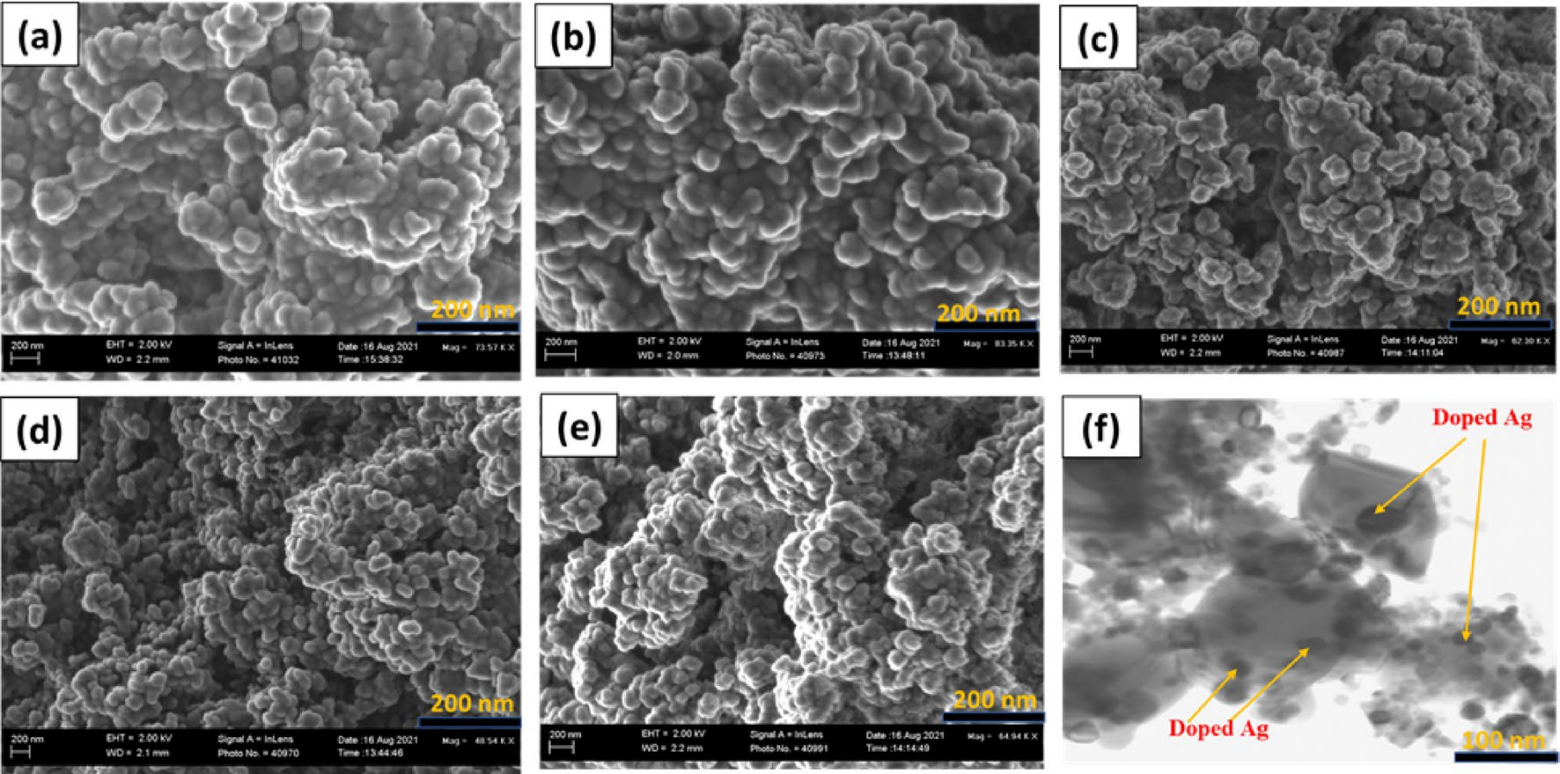
SEM images of pure ZnO (**a**) Ag–ZnO nanocomposites with (**b**) 1%, (**c**) 3%, (**d**) 5% (**e**) 8% Ag and TEM image (**f**) 8% Ag.

**Figure 5:**
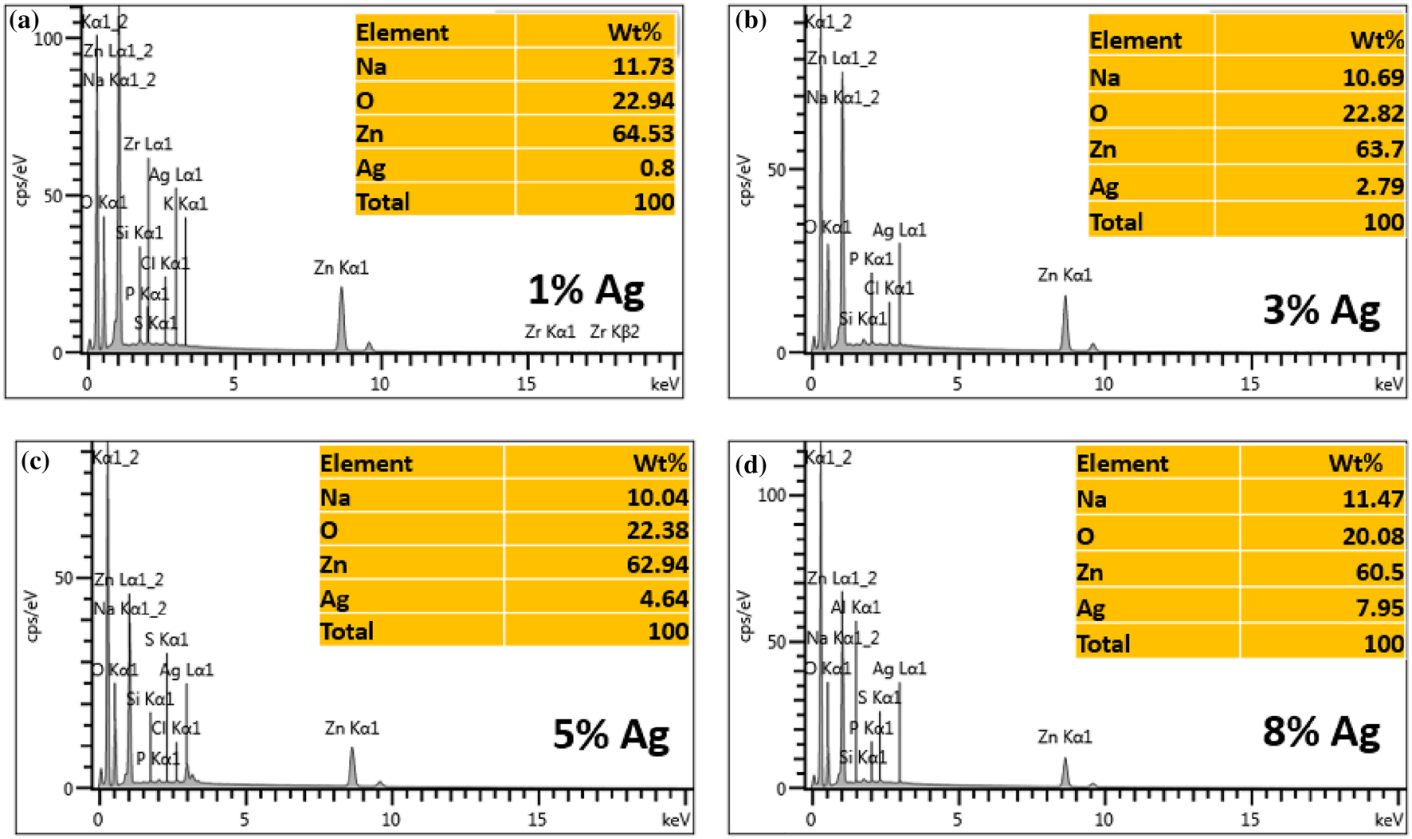
EDX spectra of (**a**) 1%, (**b**) 3%, (**c**) 5%, and (**d**) 8% Ag–ZnO nanocomposites.

Figure 4a–e shows the FESEM images of synthesized nanomaterials in which pure ZnO presented spherical nanoparticles with an average size of 64.00 nm. Furthermore, FESEM analysis showed a denser spherical-shaped Ag–ZnO nanocomposite with an average size of 76.5, 75.8, 74.6, and 47.9 nm at 1, 3, 5 and 8% Ag content, respectively. This corresponds with the XRD data, which depicts a decrease in particle size with Ag loading. On the other hand, to confirm the coexistence of Ag on ZnO nanoparticles, TEM analysis was performed on the nanocomposite with 8% Ag. The TEM image in Fig. 4f shows a dispersion of the spherical Ag nanoparticles anchored on the surface of ZnO nanoparticles, as was reported elsewhere^75^. This might be attributed to the large ionic size of Ag, which hindered the incorporation of doped Ag into the crystal lattice of ZnO^76^. The average particle size of the Ag–ZnO nanocomposite depicted by TEM analysis with 8% Ag was 12.3 nm, which is in good agreement with XRD results of the same composition as was reported elsewhere^77^. Figure 5 shows the EDX spectra for the Ag–ZnO nanocomposite in which Ag, O, and Zn signals were observed; this confirms the anchoring of Ag on ZnO on the surface. Furthermore, to ascertain the amount of Ag doped on ZnO at various concentrations, EDX analysis of Ag–ZnO with different Ag content was performed. The results of Fig. 5 show that the amount of doped Ag (1, 3, 5, and 8%) corresponds with the elemental analysis shown in the table (inset), indicating that Ag was successfully doped on ZnO. The presence of elemental sodium in the EDX spectra is ascribed to the NaOH used for pH adjustment during the synthesis of the nanomaterials.

### Effect of temperature on the green synthesis of nanomaterials

**Figure 6:**
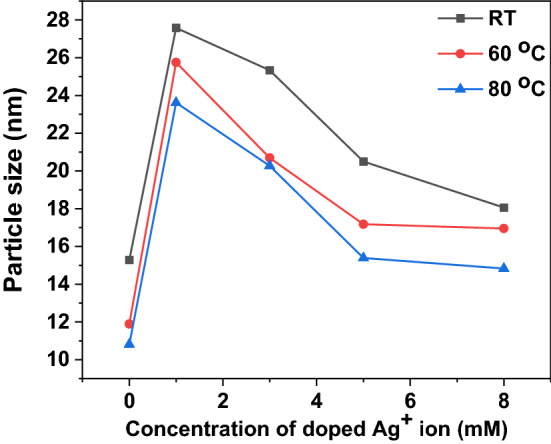
Variation of particles size for the pure ZnO and Ag–ZnO with different concentrations of Ag at different synthesis temperatures.

In wet chemistry synthesis and engineering of nanomaterials, temperature has a significant influence on the synthesis of nanoparticles through nucleation and growth of nanoparticles. The formation of nuclei (nucleation) that yields smaller particles is favored at a higher temperature, while growth is favored at a lower temperature^67,78^. In this study, the effect of temperature on the size of the synthesized ZnO and Ag–ZnO nanoparticles was investigated at room temperature (30 °C), 60 and 80 °C. Results shown in Fig. 6 show that the size of particles becomes smaller with an increase in reaction temperature. This might be attributed to the high nucleation of metal ions, because an increase in temperature impacts more nucleation than the growth of nuclei^79,80^. Normally, at high temperature, the kinetic energy of the molecules increases, and precursors are consumed more quickly to form nuclei, thus suppressing particle growth^78^. On the other hand, at lower temperatures, the kinetic energy of nanoparticles decreases. This results in crystal growth attributed to Oswald ripening^81^. As a result, at higher temperatures, smaller particles with a more uniform size distribution are formed^78^. However, when the temperature is too high, the surface activity of the nuclei is increased, which fosters the collision and agglomeration of nuclei^79^.Therefore, temperatures above 80 °C might lead to larger particle sizes^67,79^. Similar findings have been reported elsewhere^67,78^.

### Effect of concentrations of the precursor salts

The effect of the concentration of precursors on the size of Ag–ZnO nanocomposite and the efficacy of the synthesis method was studied at different concentrations of silver ions (1, 3, 5, 8 mM) doped on Ag–ZnO nanocomposites. The formation of Ag–ZnO occurs in two stages: the first stage is the generation of nuclei, followed by the growth of nuclei. Results from Fig. 6 show the decrease in size of Ag–ZnO nanocomposites with an increase in concentrations of silver salts from 1 to 8 mM. This might be ascribed to the higher nucleation of ions at higher concentrations of precursor salts, which yields smaller sized nuclei^79^. However, at higher concentrations of precursor salts, an excess number of nuclei forms, which results in agglomeration of nuclei and growth of particles^82^. A similar phenomenon was also reported in the literature^79^, where an increase in the size of green synthesized copper nanoparticles was observed when the concentration of the precursor salts increased from 7.5 to 10 mM. In this study, a maximum concentration of 8 mM for Ag salt resulted in the smallest nanocomposite. This is close to the optimal value of 7.5 mM for copper salt reported in the literature^79^.

### Effect of pH

In the green synthesis of nanomaterials, the reducing and capping of nanoparticles depend on the charge of phytocompounds, which is affected by a change in pH^79^. pH variation affects the formation and morphology of nanoparticles. Herein, the formation of ZnO nanoparticles and Ag–ZnO nanocomposites was evaluated at acidic and slight basic conditions of the *Tetradenia riperia* leaves extract. It was observed that the pH of *Tetradenia riperia* leaves extract was 5.10, but was lowered to 4.46 after the addition of metal precursors, which might be ascribed to the release of H^+^ ions by some *Tetradenia riperia* phytocompounds when they are oxidized in the presence of metal precursor ions^83^. This can be evinced from the chemical reaction of phenols with metal ions, which results in oxidized phenol, reduced metallic elements and hydrogen ions that account for the low pH of the medium. Due to the low pH (acidity) of the TR extract, the formation of nanoparticles in the medium was suppressed, which might be attributed to the inactivation of the phytocompounds^79,82^ responsible for reducing and capping of metal precursors. However, when the pH was higher (7–8), the formation of nanoparticles was observed. A similar scenario was also reported in the literature^79^ where the repression in nanoparticles formation was observed at pH of 4.7, but at pH 6.6, copper nanoparticles were observed to form from *Azadirachta indica* leaves extract. Interestingly, even a high pH was found to be effective in the formation of nanoparticles. However, agglomeration results in large-sized nanoparticles^78^. Therefore, the optimal pH for the bio-route formation of small-sized nanoparticles might be in the neutral to slightly alkaline range.

### Antibacterial activity of ZnO and Ag–ZnO nanocomposites

**Figure 7:**
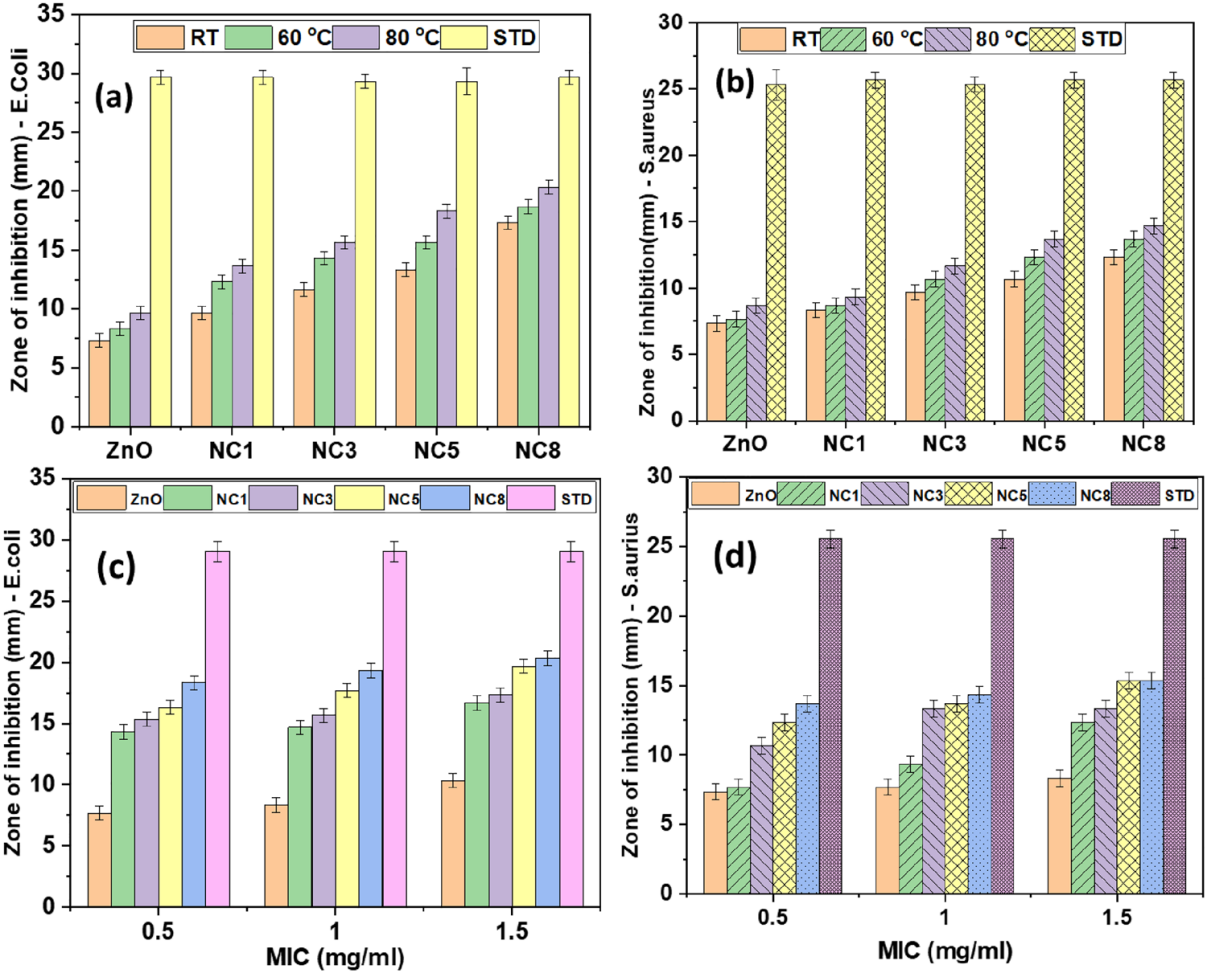
Zone of inhibition for pure ZnO and Ag–ZnO nanocomposite with different Ag content at different synthetic temperature for (**a**) *Escherichia coli* (**b**) *Staphylococcus aureus* and minimum inhibitory concentration (MIC) values for pure ZnO and Ag doped ZnO for (**c**) *Escherichia coli* (**d**) *Staphylococcus aureus*.

**Figure 8:**
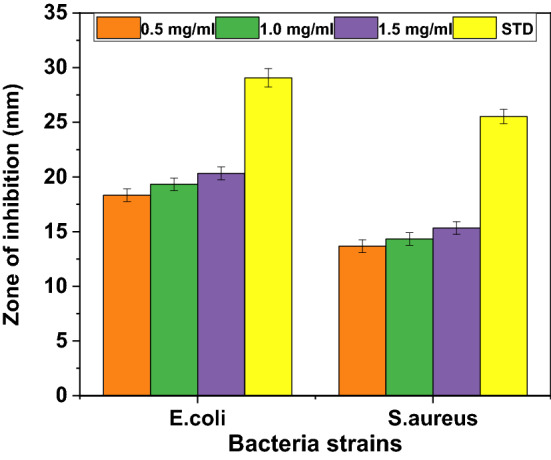
Antibacterial activity of synthesized Ag–ZnO against *Escherichia coli* and *Staphylococcus aureus* at different minimum inhibitory concentration (MIC).

**Figure 9:**
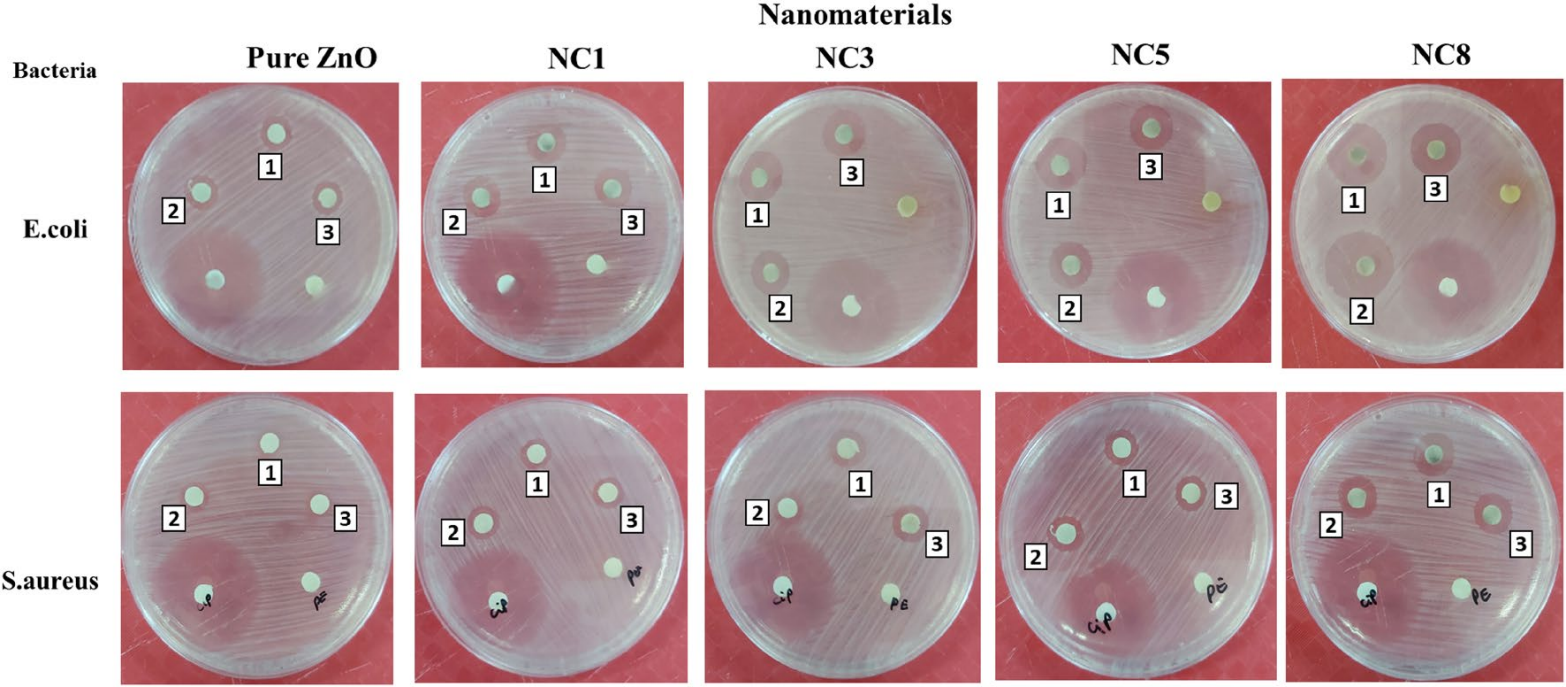
Real images showing the antibacterial activity of biosynthesized pure ZnO and Ag–ZnO nanocomposites with different Ag content (1%, 3%, 5%, and 8% Ag) against *Escherichia coli* and *Staphylococcus aureus*.

The antimicrobial activity of the biosynthesized ZnO nanoparticles and Ag–ZnO nanocomposites against *E. coli* (gram-negative) and *S. aureus* (gram- positive) bacteria strains were evaluated by determining the zone of inhibition (ZOI) and minimum inhibition concentration (MIC). Results from Table 1 and Fig. 9 show that the ZOI values of the Ag–ZnO nanocomposites are undeniably higher than for pure ZnO for both strains. This demonstrates the higher antibacterial activity of Ag–ZnO nanocomposites over ZnO nanoparticles, which is attributed to their synergic effect. Furthermore, the antibacterial activity of Ag–ZnO nanocomposites was found to increase with the increase in Ag concentrations (Fig. 7a,b. This demonstrates that doping of Ag in ZnO improves the antibacterial activity of ZnO nanoparticles. This phenomenon can be ascribed to the stronger antimicrobial effect of Ag^84,85^ as well as the smaller size of the formed nanocomposite as demonstrated by XRD and FESEM results. Furthermore, Fig. 7a,b shows that the effectiveness of nanocomposites was influenced by the temperature, in which ZnO nanoparticles and Ag–ZnO nanocomposites synthesized at RT had a moderate effect (low ZOI) compared to those synthesized at 80 °C. This might be attributed to the high nucleation rate and the formation of smaller particle sizes at 80 °C. Moreover, results from Fig. 7c,d show that the antimicrobial activity of the biosynthesized ZnO and Ag–ZnO nanocomposites, increases with MIC values. This might be attributed to the increased dose per bacteria strain, which results in high antimicrobial activity. The maximum ZOI values recorded by Ag–ZnO were 19.33 ± 0.58 mm for *E. coli*, and 14.33 ± 0.58 mm for *S. aureus* whereas ZnO recorded lower ZOI values of 8.33 ± 0.58 mm and 7.67 ± 0.58 mm for *E. coli* and *S. aureus* respectively. Similar results were reported elsewhere^6,43^. These results demonstrate that the incorporation of Ag in ZnO nanoparticles improves the antibacterial activity of the Ag–ZnO nanocomposite due to a synergetic effect. Therefore, this provides insight that E. coli is more susceptible to Ag–ZnO nanocomposites (Fig. 8). This might be attributed to differences in the cell wall composition of the two bacteria^43,48,86^, in which the cell wall of *S. aureus* bacteria is covered by a thick and rigid peptidoglycan layer crosslinked by peptide chains, which limits the penetration of the Ag–ZnO nanocomposite^85^. On the other hand, the cell wall of E.coli contains a thin peptidoglycan layer, facilitating easy penetration of Ag–ZnO nanocomposites^86^. Similar findings have been reported elsewhere^43,86^. Various approaches have been adopted for the synthesis of ZnO NPs and Ag–ZnO nanocomposites using green methods. It is shown in Table 2 that nanomaterials from this study show better performance when compared to concentrations used in other studies.

**Table 1:** Antibacterial activity of the synthesized nanomaterials using agar disc diffusion method at a MIC of 1.5 mg/ml.

S/N	Bacteria strain	Zone of inhibition (mm)					
		Standard antibiotic disc^a^	Pure ZnO	NC1	NC3	NC5	NC8
1	*Escherichia coli*	29.07 ± 0.84	8.33 ± 0.58	14.67 ± 0.58	15.67 ± 0.58	17.67 ± 0.58	19.33 ± 0.58
2	*Staphylococcus aureus*	25.53 ± 0.66	7.67 ± 0.58	9.33 ± 0.58	13.33 ± 0.58	13.67 ± 0.58	14.33 ± 0.58

^a^Ciprofloxacin.

**Table 2:** Comparison of antimicrobial activity of ZnO and Ag–ZnO from this study with other published data.

Name of bacteria	Antimicrobial agent	Synthesis methods	Plant extract	Crystallite size	Concentration (MIC)	Zone of inhibition (mm)	References
*Staphylococcus aureus*	ZnO	Green synthesis	*Suaeda aegyptiaca*	60 nm	10 mg/ml	16.01	^89^
*Staphyococcus aureus*	Ag–ZnO	Hydrothermal	*Trigonella foenum-graecum*	75 nm	20 mg/ml	13.5 ± 0.707	^18^
*Escherichia coli*	Ag–ZnO	Hydrothermal	*Trigonella foenum-graecum*	75 nm	20 mg/ml	12.5 ± 0.707	^18^
*Staphyococcus aureus*	ZnO	Hydrothermal	*Psidium guajava*	12 nm	100 mg/L	28.1 ± 0.1	^27^
*Escherichia coli*	ZnO	Hydrothermal	*Psidium guajava*	12 nm	100 mg/L	26.7 ± 0.2	^27^
*Escherichia coli*	Ag–ZnO	Precipitation	*Padina gymnospora*	31.2 nm	200 μg/mL	19.5 ± 0.2	^47^
*Staphyococcus aureus*	Ag–ZnO	Precipitation	*Padina gymnospora*	31.2 nm	200 μg/mL	18.1 ± 0.1	^47^
*Staphyococcus aureus*	ZnO	Green synthesis	*Thymus vulgaris*	NIL	20 μg/mL	12 ± 0.21	^48^
*Escherichia coli*	ZnO	Green synthesis	*Thymus vulgaris*	NIL	20 μg/mL	11 ± 0.24	^48^
*Staphyococcus aureus*	ZnO–Ag	Green synthesis	*Thymus vulgaris*	5.0 nm	20 μg/mL	18 ± 0.24	^48^
*Escherichia coli*	ZnO–Ag	Green synthesis	*Thymus vulgaris*	5.0 nm	20 μg/mL	15 ± 0.21	^48^
*Escherichia coli*	ZnO	Green synthesis	*Aloe socotrina*	15–50 nm	100 μg/mL	25.3 ± 1.7	^21^
*Staphyococcus aureus*	ZnO	Green synthesis	*Azadirachta indica*	96 nm	1 mg/ml	45 ± 0.37	^21^
*Escherichia coli*	ZnO	Green synthesis	*Azadirachta indica*	96 nm	1 mg/ml	48.5 ± 0.38	^21^
*Staphyococcus aureus*	ZnO	Green synthesis	*Tetradenia riperia*	10.8 nm	1.5 mg/ml	7.67 ± 0.58	This study
*Escherichia coli*	ZnO	Green synthesis	*Tetradenia riperia*	10.8 nm	1.5 mg/ml	8.33 ± 0.58	
*Staphyococcus aureus*	Ag–ZnO	Green synthesis	*Tetradenia riperia*	14.8 nm	1.5 mg/ml	14.33 ± 0.58	
*Escherichia coli*	Ag–ZnO	Green synthesis	*Tetradenia riperia*	14.8 nm	1.5 mg/ml	19.33 ± 0.58	

### The mechanisms of antibacterial activities of Ag–ZnO

The Ag–ZnO nanocomposites attack and cause bacterial cell lysis by several mechanisms. Firstly, the interaction of smaller-sized nanoparticles with the bacterial cell leads to membrane penetration due to disruption of the cell membrane caused by changes in membrane protein or enzyme activity^87^. This allows the entry of Ag and ZnO nanoparticles into the bacteria cell and results in the defacement of the lipid bilayer and membrane protein. This in turn causes an imbalance within the cell, which leads to cell death. Secondly, surface oxidation of the Ag–ZnO nanocomposite results in the release of silver (Ag^+^), which influences the electrostatic interactions between the ions formed and the negatively charged bacterial cell wall. This results in the high antibacterial activity of the Ag–ZnO nanocomposites reported in this paper on E. coli, as was reported elsewhere^43^. Thirdly, due to the penetration of the nanoparticles inside the bacteria cell, trickling of cytoplasm occurs; this results in shrinkage of the cell membrane and death of bacteria^6^. Fourthly, Ag–ZnO nanocomposites hinder the replication of DNA by releasing Ag^+^, which interact with sugar-phosphate groups, thus mutating the gene and affecting the cellular functioning of bacteria^6,88^. On the other hand, illuminated ZnO nanoparticles produce reactive oxygen species (ROS) that lead to oxidative stress in the cell^89^. This affects mitochondrial activities, weakening the metabolic activities, which ultimately lead to cell death. When ZnO nanoparticles or its generated ROS obstruct the signal transduction pathway, vital cell functions such as DNA replication, transcription, and translation are halted, resulting in cell death.^18,27,89^. Therefore, the aforementioned mechanisms show that nanocomposites offer multitarget mechanisms for denaturing the bacteria strains. This suggests that Ag–ZnO nanocomposites may have superior antibacterial activities when compared to conventional antibiotics or disinfectants (Fig. 9).

## Conclusion

The Ag–ZnO nanocomposites were successfully synthesized through an environmentally benign approach by using an aqueous leaf extract of Tetradenia riperia plant and were evaluated for their antimicrobial activity against *E. coli* and *S. aureus* bacteria strains. The SEM and XRD analysis revealed that biosynthesized Ag–ZnO nanocomposites were spherical and crystalline in nature and were observed to decrease in average particle size from 23.6 to 14.8 nm as a result of an increase in Ag concentrations. Different antimicrobial activities of Ag–ZnO nanocomposites were investigated and found to have higher antimicrobial activity against *E. coli* than *S. aureus* bacteria. The Ag–ZnO nanocomposites presented higher antimicrobial properties compared to ZnO nanoparticles. This provides an insight that the addition of silver (Ag) nanoparticles improves the antimicrobial activity of the Ag–ZnO nanocomposites, especially for *E. coli* (gram-negative bacteria). Furthermore, the antimicrobial activities of Ag–ZnO were found to increase with increasing Ag dopant concentrations and synthetic temperatures, indicating that smaller particles form at higher Ag concentrations and temperatures. In this regard, the biosynthesized Ag–ZnO nanocomposite provides promising results for the application in various environmental health fields, such as water disinfection.

## Data Availability

The datasets generated and/or analysed in this study are available in the DRYAD repository, https://datadryad.org/stash/share/n4Y_Pm0gXObU_5ngMAfCDNUqQbU_4OFqng1RtXt1sXQ.
